# Internal Urethrotomy, Etc.

**Published:** 1893-07

**Authors:** 


### INTERNAL URETHROTOMY.

AN ABSTRACT OF THE RESULT OF THIRTY-SIX OPERATIONS. BY B. MERRILL RICKETTS, M. D., PH. B., CINCINNATI, OHIO.

The operation of itself I consider a simple one, and if sepsis is guarded against, febrile disturbance will not occur. The complications that arose in these thirty-six cases were:

1. Urethral fever.
2. Orchitis.
3. Perforation into the rectum.

Of the first, I have had three cases where the temperature reached 102° and which subsided within seventy-two hours. We know that the simple introduction of a catheter or sound, hard or soft, will cause a rise in temperature which is usually of short duration, and we have reason to believe that this rise of temperature is due to septicæmia.

In the second class—orchitis—it is interesting to note that inflammation of the testicle did not occur until after the eleventh day in one, thirteenth in another, and fourteenth in another. The eleventh began while the patient was still confined to his room. The thirteenth began four days after the patient had returned to his duties, which were those of a commercial traveler. This great length of time I believe will exclude the primary operation as being the cause of the orchitis.

In two of the cases—the first and the second—there was no introduction of the sound after the withdrawal of the urethrotome. In the third, however, the sound was introduced every third day; possibly the introduction of the sound in this case caused the orchitis.

In the third—perforation into the rectum—I feel that there is a ray of hope of my not being responsible when we consider the possibility of there having existed a recto-urethral fistula.

The urethra was serpentine and only admitted a No. 17 French sound with great difficulty. I advised an internal urethrotomy, and my attempt to do the operation under the influence of chloroform was thwarted. The patient was allowed to come from under the influence of the chloroform and informed of the difficulty. On the next day I was successful, but, before adjusting the instrument with my finger in the rectum, I found that its point and my finger came together. The bladder was entered without difficulty, the operation done, and a No. 30 French sound introduced with ease, and the patient returned to his work, that of a book-keeper, the following Saturday.—*N. Y. Med. Jour., St. Louis Clinique.*

---

CAMPHORIC ACID IN THE NIGHT SWEATS OF PHTHISIS.—A study of the charts of cases cited does not show any appreciable change in temperature, pulse or respiration, that can be attributed to the influence of the drug. Beyond the relief of a very disagreeable and distressing symptom, the drug seems to be entirely without influence over the course of the disease. In nearly every case a small dose (10 grains) was given first. This dose, though it usually diminished the sweating, was not sufficient to entirely control it. Larger doses (20 grains) were followed by very satisfactory results. It is striking that, when once the system was under the influence of the drug, sweating did not recur for some time, and that then smaller doses, given at irregular intervals, were sufficient to hold the perspiration in check. In some cases after several doses there was a complete absence of sweating for some time. No gastric disturbance was noticed or any other disagreeable symptom. The only secretory glands affected were the sweat glands. As far as could be determined the drug is perfectly harmless and its administration is not followed by any deleterious action upon the system.

W. T. HOWARD, M. D.,
*In The American Therapist.*

---

FOR SUMMER DIARRHŒA OF NURSING INFANTS.—Toussaint has proved the excellent results by the use of the following mixture in the summer diarrhœas of nursing infants and those artificially fed:

| | | |
|---|---|---|
| ℞. | Papain pur., | gr. ix. |
| | Acid. lactic., | ℨ ss. |
| | Syr. simp | f ℥ iss. |
| | Aq. destillatæ | f ℥ v. |
| | Tr. vanillæ | q. s. |

The milk given to the patients is to be limited in quantity, and the intervals between feeding lengthened. After each nursing a coffee spoonful of the mixture is to be administered. The green stools are soon corrected, and the dyspeptic symptoms relieved.—*Gazette de Hopitaux*, October, 1890.

---

ON PLUGGING THE CERVIX UTERI INSTEAD OF VAGINA IN UTERINE HÆMORRHAGE.—Plugging the cervical canal in cases of severe uterine hemorrhage (other than post-partum) will be found more effectual and much more comfortable to the patient than the older method of plugging the vagina. The want of a simple means for completely filling the cervical canal has long been felt, but I think it will be found a simple matter by the use of an instrument constructed as follows: It is simply an improved form of sliding depositor, the difference being that, instead of the canula being pushed forward on the rod, the latter is made to retreat into the canula by the pressure of the fingers on projecting side-bars, and so the cotton plug dressing on the top of the rod is pushed home with certainty into the cervix. It may be urged that in cases of metorrhagia or very early abortion a sponge-tent or sea-tangle would be an easier mode of treatment, and quite as eflectual. But besides the inconvenience of having to place a tampon in the vagina as well, to insure retention of the tangle or sponge, we have also the great liability to sepsis, and I certainly prefer being on the safe side. The size of the os being determined by digital examination, a piece of absorbent cotton is to be neatly wound round the projecting point of the instrtument for about two inches of its length, and thickly enough to fully fill the canal.

The cotton plug is then to be saturated with whatever antiseptic styptic the operator prefers—saturated solution of alum in glycerin, iodine tincture or turpentine; but I have a strong dislike for iron. A large sized cylindrical speculum is then introduced and the os and cervix syringed with hot water, so as to wash away all clots. The cervix is then to be steadied with a tenaculum, held in the left hand, while the plug is passed into the cervix, with the depositor held in the right, as far as possible, and on pressure being made on the side-bars while the thumb supports the base of the instrument, the plug is pushed off the rod and the depositor withdrawn. The whole operation can be done without assistance. If the plug has been made of the proper dimensions, which, of course, must depend upon the judgment of the operator, and inserted fully into the cervical canal, there need be no anxiety as to the result, and the antiseptic and astringent action of turpentine, which I generally use myself, leaves nothing to be desired. The plug may be safely left till expelled by uterine action, when, if the hæmorrhage continue, the practitioner will be prepared to adopt other measures––washing out or curetting the uterus, or both. With a sponge-tent or sea-tangle in the womb it is always an anxious time for the practitioner, but with the plug properly in position he can take his own time about further interference.––*Med. and Sur. Reporter.*

---

THE BEST METHOD OF STERILIZING HARD AND SOFT RUBBER INSTRUMENTS.–Lannelongue (Goz Hebdomadaire, 1892,) recommends for the sterilizing of hard and soft rubber instruments, subjecting them to the fumes of quicksilver. The instruments are rolled in flannel impregnated with mercury and placed in closed vessels. By a simple test you can demonstrate that the space becomes filled with fumes of quick-silver. The bacteriological examinations show that after a very short time the intruments are thoroughly aseptic. As a lubricant, the author is in the habit of using olive oil; a few globules of the quicksilver are kept on the bottom to keep the oil sterile. Since these measures have been adopted the author has had the best results.—*Universal Medical Magazine.*

### ON TREATMENT OF BUBO.

BY F. S. WATSON.

This paper is a plea for the treatment of bubo by excision, and the subsequent attempt to obtain primary union.

Twenty-two buboes were treated, and in ten, a little less than one-half, perfect union by first intention resulted.

As regards the technique of the operation, the most important points are as follows:

1. To remove thoroughly all diseaseed tissue, and to leave, as far as possible, a perfectly healthly surface in every part of the wound.

2. To remove all portions of the skin, which are, or threaten to become, necrotic.

3. To curette the under surface of the skin flaps.

4. To thoroughly swab the whole surface with dry sterilized gauze or sponges wet with a solution of corros. subl. 1:4000.

5· To tie, as far as possible, the end of all lymph vessels that have been divided.

The author considers this last point of especial importance, and one which has not hitherto been attended to. If not ligated, these vessels pour into the wound a large amount of fluid which lifts up the flap and lessens the chance of primary union. These vessels closely resemble cutaneous nerve branches, but are distinguished from them by having a distinct lumen, from which clear fluid exudes, and are of a more yellowish color, and present a duller surface.—*Jour. Cut. and Ver. Dis. Ex.*

---

THE PRESERVATION OF VACCINE LYMPH. — According to Chambon and Menard, vaccine lymph, when combined with glycerin and placed in sterilized tubes, improves with age, although when used in the fresh state it is found to contain micro-organisms (staphylococcus, etc.) The parasitic microbes not only diminish in number when the lymph is kept for a period of sixty days, but ordinary lymph improves and produces a more typical eruption.—*Ex.*

### "THE ACTION OF PHYTOLINE IN OBESITY."

BY I. N. LOVE, M. D., ST. LOUIS.

The statement will be accepted by all, that fat is not the highest grade of tissue ; its object in the anatomy being really as a packing material, externally serving for the artistic purpose of rounding out the lines of the figure in a manner to favor curves and that which is graceful rather than angles and abrupt prominences.

Fat, in proper quantity, favors comfort and favors the really beautiful, and smoothes out the tangled threads of care and the wrinkled lines left by carking worries, and the fuming and fretting of the every-day annoyances of life. It does not exist very much as a necessity to the physique, but rather as an accessory. That there can be too much of a good thing, goes without saying, and the numerous mountains of flesh moving about our streets and hiding themselves in their homes, will give evidence in favor of the truth of this statement.

An excessive amount of fat is not only unsightly, but is unhealthy ; in fact, as an evibence favoring the thought that fat is a low grade tissue, we speak of other tissues degenerating into fat. Certainly, the tendency toward the accumulation of an extra and unnecessary amount of fat favors a dangerous fatty degeneration of the heart and the tissues forming other important organs.

The proper selection of diet, with exercise, can do much toward the diminishment of fat ; but the profession and the laity have long looked for some remedy which could be depended upon to assist toward this consummation, devoutly to be wished. In Phytoline we have such a remedy ; this remedy being prepared from the active principle of the berries of the Phytolacca Decandra, after having been touched by the early frost. The remedy has been used for rheumatism for many years, and also for the diminishment of excessive glandular growths, and also as a helper for the drying-up and the reducing in size of the breasts of nursing women. Inflammation and abscess of the manuary glands have often been prevented by its use.

The reason for its acting favorably in these conditions, have not been given. Clinically the results have been satisfactory. In a number of cases wherein I have directed the using of the Phytoline, for the reduction of fat, I have had favorable results, and I am persuaded that the profession is justified in the use of the medicament. In the several cases wherein used, ten-drop doses were given both before and after the three daily meals, making six doses per day. The clinical reports are satisfactory. Even though as yet administering this remedy empirically, I feel that the uniformily favorable clinical results justify strong hopes from its use.

I have observed nothing objectionable in its application, and shall continue to watch results with interest.

---

### TWO UNUSUAL CASES OF TRACHEOTOMY.

BY C. G. JENNINGS, M. D., DETROIT.

CASE I.—*Scarlatina; Severe Cervical Adenitis; Laryngeal Stenosis; Tracheotomy; Recovery.*

On January 20th, at six o'clock a. m., I was called by Dr. H. R. Tilton, U. S. Army, to see Miss M. S., aged eighteen years. For some months previous to this time she had been affected with chronic naso-pharyngeal catarrh. On January 12th, she became acutely ill with sore throat and fever. On the 13th the temperature was 103.8° with a scarlatina rash well developed on the body. The scarlet fever ran a moderate course, the temperature never rising above the point it reached on the second day. On January 17th, the cervical glands began to swell and the left ear to discharge. On the 19th the glands and connective tissue on the left side of the neck were very much swollen. Dr. Tilton was called at midnight of the 19th on account of the severe dyspnœa that had suddenly developed. The doctor noted that expiration was free but inspiration prolonged and difficult. The glands of both sides of the neck had continued to enlarge and the surrounding connective tissue so involved that the whole neck was enormously increased in size. Hot applications gave some relief at first but the dyspnœa soon returned and continued to increase in severity. When I saw her at six o'clock on the following morning the dyspnœa was quite severe and the patient complained of the great distress it caused her. Examination of the throat with the laryngoscope showed the pharynx intensely red and œdematous, the tonsils swollen, and the whole throat covered with small flakes of pseudo-membrane. The great swelling and dyspnœa prevented a satisfactory inspection of the larynx. The epiglottis, however, was seen to be œdematous and pushed over toward the right side by the greater swelling on the left. The dyspnœa at this time was not great enough to demand surgical interference. The hot applications externally, and a steam spray internally, were used with vigor. The dyspnœa, however, continued to increase. At 12 o'clock on the 20th it had become so severe that interference was thought best. I first attempted an intubation. The patient was placed upright in a straight-backed chair and bound securely by a sheet, her head being supported by Dr. Tilton and the nurse. The swelling of the pharynx prevented the use of the laryngeal mirror, but the age of the patient was such that the epiglottis was just within reach of the index finger. The tube was quickly passed, but the swelling was so great and the canal so distorted that the tube failed to enter the larynx. This first attempt precipitated an alarming attack of dyspnœa, and in a moment the larynx closed and respiration ceased. I quickly made three or four more attempts to find the larynx but failed. A convulsion came on and apparent death supervened. Tracheotomy instruments were at hand and I quickly seized a knife and with two or three passes opened the trachea just above the sternal notch. The tracheal wound was held apart by retractors and a soft catheter introduced well down into the trachea and through it I inflated her lungs from my own. Expiration was produced by pressing the walls of the chest. This was repeated about a dozen times when a voluntary inspiration took place and very soon the patient was again breathing. A second convulsion occurred just as the respirations were beginning, and an involuntary evacuation of urine and fæces took place at the same time. After lying quietly for a few minutes the patient opened her eyes and consciousness quickly returned. A hypodermatic injection of digitalin and strichnine was given and the patient put to bed, surrounded by hot bottles. The reaction was prompt.

The cervical swelling continued to increase and on the 22d pitting upon pressure was noted on the left side. Dr. T. A. McGraw had been added to the consultation, and, under chloroform, he cut down on the anterior border of the sterno-mastoid and found pus at the depth of two and one-half inches. On the 26th the larynx permitted a small amount of air to pass through, and a smaller tracheal tube was introduced. On the 27th signs of a deep collection of pus showed upon the right side. Dr. Tilton made an incision corresponding to the one upon the left side, and found pus at the depth of two inches. The pharyngeal and laryngeal swelling now rapidly subsided, and the tracheal tube was removed on the 28th. From this time convalescence was rapid. The tracheotomy wound and the abscess openings promptly healed. Desquamation was complete on the 22d of February.

CASE II.—*Whooping Cough; Laryngeal Diphtheria; Tracheotomy; Recovery.*

Della V., age five years, in the third week of a severe attack of whooping-cough developed a mild pharyngeal diphtheria. On the third day the disease invaded the larynx, and on the fifth day the stenosis was so great as to demand operative interference. At 9 o'clock p. m., I opened the trachea above the thyroid isthmus. The operation was one of great difficulty on account of the fact that every incision precipitated a violent paroxysm of coughing, and suffocation was repeatedly threatened. After a thorough cleansing of the windpipe the tube was inserted and the patient quickly rallied, The paroxysms of coughing disappeared almost entirely after the operation, no membrane reformed, and the patient made a rapid convalescence, the tube being removed on the twelfth day.

These two cases of tracheotomy are interesting; the first from the fact that cervical adenitis in scarlatina, although often very severe and causing great glandular and connective tissue swelling, rarely produces stenosis so severe as to demand operative interference. I have met with three cases before this one that assumed very threatening symptoms, but all of them recovered without operation. The second is interesting as a case of tracheotomy for diphtheria during the paroxysmal stage of whooping-cough. I selected tracheotomy in preference to intubation because of the presence of pertussis. I thought the chances of the child retaining the intubation tube during the violent coughing paroxysms to be very small. It is also interesting to note the almost entire absence of the paroxysmal coughing after the operation, and even after the removal of the tube and re-establishment of laryngeal respiration no paroxysmal coughing occurred.—*Archives of Pediatrics*, *June*, 1893.

### TREATMENT OF DISEASE OF INEBRIETY.

A meeting of the American Association for the Study and Cure of Inebriety, held at the New York Academy of Medicine, March 23, 1893, Dr. Edward C. Mann, of Brooklyn, N. Y., Medical Superintendent of Sunnyside Private Hospital for diseases of the nervous system, alcoholism and the opium habit, read a paper on "Science vs. Folly in the Treatment of Disease Caused by the Abuse of Stimulants and Narcotics: A Plea for the Suppression of the Nostrum, Patent Medicine and Specific in Rational Therapeutics." After comparing scientific medication with charlatanism and showing the physiological action of alcohol on man and his offspring, as well as the diseases produced by indulgence, Dr. Mann passed to the subject of the treatment of disease of inebriety. He recommended the following as a good tonic and sedative in dipsomania, having a good effect on the stomach, and tending to antagonize both the degenerative changes in the brain, and the effects of alcohol on the structures of the body:

℞ Quininæ sulph........................................grs. ii.
Zinc oxide........... ..................................grs. ii.
Strychnia sulph....................................gr. 1-40.
Arsenic ..... .........................................gr. 1-500.
Capsicum ...............................................grs. ii.

M. et ft. pill No. i. Sig.—One pill three times a day.

Together with this pill, Dr. Mann uses in his private hospital for sixteen days the following hypodermatic dosiometry:

℞ Strychnia nitrat....................................gr. i.

Aquæ dest..............................................ʒ ss.

M.—Eight minims daily for eight days; 4 minims daily for another eight days. To quiet the morning nausea of alcoholics, two or three drops of wine of ipecac on the tongue, fasting.

The patient is kept in bed for the first few days, and fed on milk and meat juice for nourishment. Hydrotherapy and electrotherapy are employed. To induce sleep, the following sedative is administered at night for a few days:

℞ Tr. opii deod.

Ext. hyoscy. fl.

Chloral hydrat.

Pot. bromid.

} aa.............................ʒ i.

Tr. capsici.............................................ʒ ss.

Tr. aconit. rad.......................................m v.

Aquæ menth. pip.......... .......ad...............℥ vi.

M. et Sig.—Two Tablespoonfulls at bedtime for a few days only, freely diluted with water.

If the patient is very much excited and is bordering on delirum tremens, the following is useful for two or three nights:

℞ Hyoscin. hydrobromat.. ......................... .. gr. i.

Aquæ dest.......... .................................ʒ ix.

Spt. vini rect.........................................ʒ i.

M. et ft. hypodermatic solution. Sig.—Dose from 5 to 10 minims *pro re nata.*

In order to render Dr. Mann's pill available to the medical profession, Parke, Davis & Co., have added it to their list of gelatin-coated pills, which they are now prepared to supply in bottles of 100 or 500.—*The National Medical Review.*

